# Incidence and survival differences in esophageal cancer among ethnic groups in the United States

**DOI:** 10.18632/oncotarget.16694

**Published:** 2017-03-30

**Authors:** Zheling Chen, Yinghong Ren, Xianglin L Du, Jiao Yang, Yanwei Shen, Shuting Li, Yunying Wu, Meng Lv, Danfeng Dong, Enxiao Li, Wei Li, Peijun Liu, Jin Yang, Min Yi

**Affiliations:** ^1^ Department of Medical Oncology, The First Affiliated Hospital of Xi’an Jiaotong University, Xi’an, Shaanxi, People's Republic of China; ^2^ Department of Internal Medicine, Shangluo Central Hospital, Shangluo, Shaanxi, People's Republic of China; ^3^ Department of Epidemiology, Human Genetics and Environmental Sciences, The University of Texas School of Public Health, Houston, TX, USA; ^4^ Weapon Industry Health Research Institute, Xian, Shaanxi, People's Republic of China; ^5^ Department of Translational Medicine, The First Affiliated Hospital of Xi’an Jiaotong University, Xi’an, Shaanxi, People's Republic of China; ^6^ Department of Breast Surgical Oncology, The University of Texas MD Anderson Cancer Center, Houston, TX, USA

**Keywords:** esophageal cancer, the united states, survival, incidence rate, ethnicity

## Abstract

**Objectives:**

This study was performed to identify the differences in incidence, clinicopathological features, and survival in esophageal cancer among ethnic groups in the United States and to determine the reasons for the differences.

**Result:**

A total of 49,766 patients were included. Black and Asian groups had a higher proportion of squamous cell carcinoma (ESCC) (85.5% and 75.4%, respectively) and mid-esophagus tumor (43.2% and 37.7% respectively) than the non-Hispanic white and Hispanic white groups. The incidences of ESCC in all ethnic groups declined since 1973, especially in black males. At the same time, incidences of esophageal adenocarcinoma (EAC) dramatically increased in white males since 1973. And incidences of ESCC and EAC were the lowest and stable in Asian female. Multivariable models showed that patients who were male, or black, or had larger tumors, or positive lymph nodes had an increased risk of death from esophageal cancer, while patients with ESCC or diagnosed after 2005 or treated with surgery had a lower likelihood of death. For ESCC, the black patients had the lowest DSS, while for EAC there were no significant differences in DSS among the ethnic/racial groups.

**Materials and Method:**

From the Surveillance, Epidemiology, and End Results Program database, patients diagnosed with esophageal cancer from 1998-2013 were identified. Differences in incidences, clinicopathological features, treatments, and disease-specific survival (DSS) in four broad racial/ethnic groups were compared.

**Conclusion:**

Histological type distribution between racial groups could be an important consideration in the incidence and the survival trend but other factors could also have an effect.

## INTRODUCTION

Esophageal cancer was the eighth most common cancer and the sixth most common cause of cancer-related death worldwide; 456,000 new cases and 400,000 deaths were estimated for 2012 [1]. Geographically, the highest-prevalence areas were distributed mainly along two belts, one from north central China through the central Asian republics to northern Iran, and one from eastern to southern Africa [2]. Previous studies have shown ethnic differences in incidence rate, mortality rate, and disease characteristics of esophageal cancer [3–5]. Histological type, cancer stage, tumor grade, and other clinical features have been found to differ significantly between ethnic/racial groups in United States. For example, black (African Americans) patients had higher rates of esophageal squamous cell cancer (ESCC), while whites had higher rates of esophageal adenocarcinoma (EAC) [6]; the uneven distribution of histological cancer types between racial groups resulted in ethnic/racial differences in the disease incidence and outcomes. The incidence rates of EAC increased rapidly in the United States from 1975 to 2001 [7], while the incidence of ESCC declined during the same period. The 5-year overall survival rate for EAC increased from 5% to 20%since the late 1970s and has exceeded that of ESCC [8]. Other factors such as tobacco use, alcohol consumption, diet, and methods of treatment also affected the incidence percentages and survival durations among racial groups [9–11]. Furthermore, immigrants, as a special population, differed from native-born residents in many disease characteristics. For example, among esophageal cancer patients in California, Asian-Americans showed different clinical characteristics than those of the NHW group, with lower percentages of EAC and higher percentages of ESCC [8].

Previous studies have indicated racial differences among ESCC patients in clinicopathological features and survival duration [12]; the EAC survival in United States adults have been reported [12, 13]. Given these racial differences and trends, a comprehensive consideration of ethnicity/race, histology, stage, and other factors is needed when analyzing the incidence of and survival outcomes for esophageal cancer. In this study, we performed a large population-based retrospective analysis to identify differences in incidence rates, clinicopathological features, treatments, and survival rates among ethnic groups in the United States and clarified the reasons for the identified differences.

## RESULTS

### Patient demographic and clinicopathological characteristics

49,766esophageal cancer patients diagnosed from 1998-2013 were included in this study: 77.1% non-Hispanic white (NHW), 11.8% Black, 6.7% Hispanic white (HW), and 4.4% Asian. The median age at diagnosis was 68 years old. The majority of patients had regional (31.0%) or distant (34.2%) disease. 61.7% patients had EAC histology, and 58.6%patients had tumor(s) located in the lower esophagus.

Table 1 shows the baseline demographic and clinicopathological characteristics for the four racial/ethnic groups. Black patients were younger at diagnosis(median age, 63years) than NHW and Asian patients (median age, 68 years for both; P=0.0001). Furthermore, 56.8% of black patients were diagnosed at ages younger than 66 years old, compared with 41.3% of the NHW, 48.7% of the HW and 40.8% of the Asian patients were diagnosed at ages younger than 66 years old (P<0.0001). The proportions of patients with cancer located in the lower part of the esophagus were higher among NHW group (73.7%) and HW group (61.3%), while the proportion of patients with cancer in the middle part of the esophagus was highest in black patients (43.2%). Asian patients were more likely to have positive lymph nodes than the other groups. Regarding histological types, 85.5% of the black group and 75.4% of the Asian group had ESCC, compared with only 28.6% of the NHW group and 42.6% of the HW group had ESCC (P < 0.0001).

**Table 1:** Baseline demographic and clinicopathologic characteristics of the 49,766 study patients

Characteristic	All patients, % (n = 49,766)	NHW, % (n = 38,378)	Black, % (n = 5,881)	HW, % (n =3,335)	Asian, % (n =2,172)	P
Age at diagnosis, years						0.0001^a^
Mean (median)	67 (68)	68 (68)	64 (63)	65 (66)	68 (68)	
<=65	43.6	41.3	56.8	48.7	40.8	<0.0001
>65	56.4	58.7	43.2	51.3	59.2	
Sex						<0.0001
Female	22.5	21.5	29.6	19.8	25.0	
Male	77.5	78.5	70.4	80.2	75.0	
Marital status						<0.0001^b^
Single	14.9	12.6	34.9	20.0	11.8	
Married	55.0	61.4	31.7	55.6	67.0	
Other	25.3	26.0	33.4	24.4	21.2	
Unknown	4.8	--	--	--	--	
Tumor grade						<0.0001^b^
I	4.8	6.1	4.9	6.1	4.7	
II	34.5	40.4	50.2	44.2	46.8	
III	41.5	51.8	44.2	48.8	47.1	
Undifferentiated	1.2	1.7	0.7	0.9	1.4	
Unknown	18.0	--	--	--	--	
SEER summary stage						<0.0001^b^
Localized	23.2	27.0	24.5	23.1	22.3	
Regional	31.0	35.0	35.5	34.0	37.4	
Distant	34.2	38.0	40.0	42.9	40.3	
Unknown	11.6	--	--	--	--	
Cancer-directed surgery						<0.0001^b^
Not performed	72.9	70.9	85.6	78.6	80.5	
Performed	26.1	29.1	14.4	21.4	19.5	
Unknown	1.0	--	--	--	--	
Lymph node status						<0.0001
Negative	35.7	53.7	54.9	50.8	47.7	
Positive	31.1	46.3	45.1	49.2	52.3	
Unknown	33.2	--	--	--	--	
Tumor location within esophagus						<0.0001^b^
Upper	8.6	8.1	18.6	11.7	17.0	
Middle	19.5	18.2	43.2	27.0	37.7	
Lower	58.6	73.7	38.2	61.3	45.2	
Overlap or location unknown	13.3	--	--	--	--	
Tumor size (cm)						0.0001
Mean (median)	5.0 (4.5)	4.9 (4.4)	5.2 (5.0)	5.3 (5.0)	5.4 (5.0)	
Year of diagnosis						<0.0001
1998-2005	43.8	43.3	48.4	40.1	44.4	
2006-2013	56.2	56.7	51.6	59.9	55.6	
Follow-up time (year)						<0.0001
Mean (median)	1.6 (0.7)	1.7 (0.8)	1.3 (0.6)	1.5 (0.7)	1.6 (0.7)	
Tumor histology						<0.0001
EAC	61.7	71.4	14.5	57.4	24.6	
ESCC	38.3	28.6	85.5	42.6	75.4	

NHW, non-Hispanic white; HW, Hispanic white; EAC, esophageal adenocarcinoma; ESCC, esophageal squamous cell carcinoma.

^a^Kruskal-Wallis equality-of-populations rank test. ^b^Calculated after exclusion of patients in the Unknown category.

We observed a heterogeneous distribution of ESCC and EAC, both in terms of tumor location and year of diagnosis. For patients with EAC histology, 88.8% of patients had tumors located in the lower esophagus, while for patients with ESCC histology, 46.4% of patients had tumors located in the middle of the esophagus (P<0.0001). During the period of 1998-2005, the diagnosis percentages for ESCC exceeded those of EAC (48.5% versus 40.8%, respectively; P<0.0001), while after 2005, the diagnosis percentages for EAC exceeded that of ESCC (59.2% versus 51.5%, respectively; P<0.0001) (Table 2).

**Table 2:** The relationship between tumor histology, location, age and year of diagnosis

Factor	Tumor histology		P value
	EAC (n = 30,705)	ESCC (n = 19,061)	
Tumor location within esophagus *			<0.0001
Upper	832 (3.1)	3,447 (21.3)	
Middle	2,199 (8.1)	7,506 (46.4)	
Lower	23,952 (88.8)	5,219 (32.3)	
Age at diagnosis, years			<0.0001
Mean (median)	67 (67)	68 (68)	
<=65	13,761 (44.8)	7,938 (41.6)	<0.0001
>65	16,944 (55.2)	11,123 (58.4)	
Year of diagnosis			<0.0001
1998-2005	12,536 (40.8)	9,236 (48.5)	
2006-2013	18,169 (59.2)	9,825 (51.5)	

EAC, esophageal adenocarcinoma; ESCC, esophageal squamous cell carcinoma.

* Tumor location was overlapping or unknown in 6,611 patients (EAC, 3,722; SCC, 2,889).

### Esophageal cancer incidence rates

**Figure 1:**
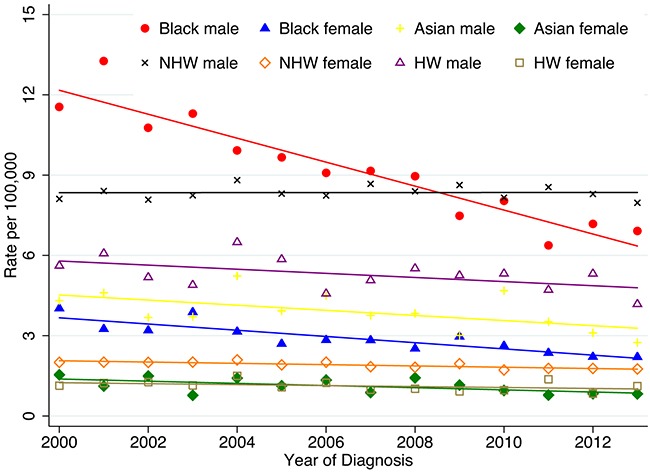
Age-adjusted SEER incidence rates by ethnicity and sex, esophagus, all ages, 2000-2013 (SEER 18 registries) Shown are invasive cases only. Rates are age-adjusted to the 2000 US standard population (19 age groups - Census P25-1130).

Figure 1 shows the age-adjusted esophageal cancer incidence rates according to ethnicity and sex for patients diagnosed from 2000 to 2013. The incidence rates in male patients were markedly higher than those in female patients in each ethnic group as well as in the population as a whole. The incidence rates in black males, HW males, and Asian males continuously decreased from 2000 to 2013, while the incidence rates in NHW males were stable. After 2008, the incidence rates in NHW males were the highest among all ethnic and sex groups. The incidence rates of Asian females and HW females were the lowest of all groups and stable since 2000.

**Figure 2:**
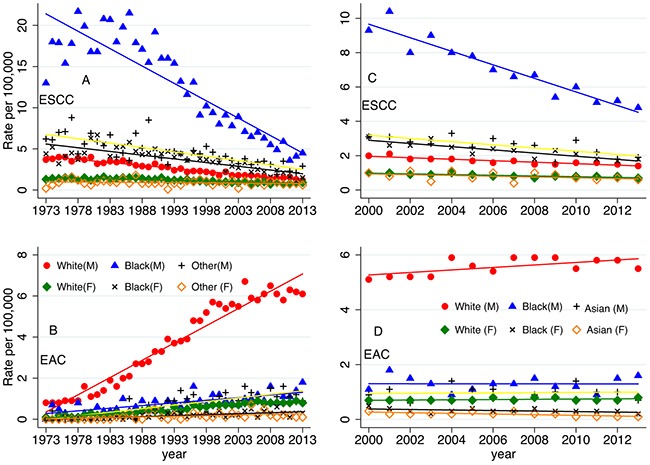
The age-adjusted SEER incidences according to race and sex from 1973-2013 (SEER 9 registries, A, B), A-esophageal squamous cell carcinoma (ESCC); B-esophageal adenocarcinoma (EAC); from 2000-2013 (SEER 18 registries, C, D), C-ESCC, and D-EAC. The percentages are per 100,000 and age-adjusted to the 2000 United States Standard Population

Besides the overall incidence trend of the esophageal cancer in different ethnic groups, further analysis about two histology types are performed as well. Figure 2 shows the age-adjusted incidence rates of different subtypes by race and sex. The incidence trends of ESCC in different ethnic groups decreased since 1973. This decrease had been particularly dramatic in black males, but it still remained higher than all other groups. Black females, white males, white females also had decreased incidence rates from 1973–2013 (Figure 2A). At the same time, the incidence rates of EAC in white males increased significantly from 1973–2013; the increasing trend was also seen in the white female and black group, but not as dramatically (Figure 2B). Since there was no Asian group in the incidence rates calculation from 1973 to 2013 period, we also included the incidence rates of ESCC and EAC from 2000 to 2013 period which included the Asian group. When analyzing the data during the period of 2000–2013 (Figure 2C&2D), the incidence rates of ESCC and EAC were the lowest and stable in Asian female. And the incidence rates of ESCC decreased in Asian males from 2000 to 2013.

### Survival

The median follow-up was 0.7 years (mean, 1.6 years; range, 0-15.9 years).

**Figure 3:**
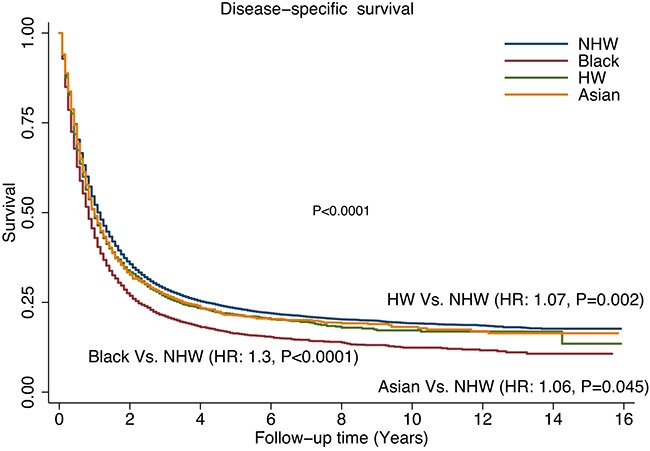
Comparison of disease-specific survival (DSS) rates by different racial/ethnic groups

Figure 3 shows the comparison of disease-specific survival (DSS) among the different racial/ethnic groups without adjustment. The black patients had a worse outcome than all three of the other groups (P<0.0001). The survival difference was especially prominent between the black and NHW groups [hazard ratio (HR) = 1.3; P < 0.0001]. Using the Cox proportional models to assess clinicopathological factors related to DSS, we first performed an analysis among all patients (Table 3). Male, older age (>65 years), poorly differentiated tumor grade, positive lymph nodes, and middle esophagus location were poor prognostic factors for DSS. Moreover, multivariable analyses confirmed that patients in the black (HR=1.19; P<00001) and HW groups (HR=1.08; P=0.03) showed independent unfavorable factors. In contrast, the ESCC histological type (HR=0.94; P=0.02), treatments with surgery (HR=0.45; P<00001), and diagnosis after 2005 (HR=0.92; P<00001) were favorable prognostic factors. Race was associated with survival in both the 1998-2005 and 2006-2013 groups. Further stratification analyses according to SEER summary stage (localized, regional, and distant) were also carried out (Table 4). When adjusted for cancer stage, the age at diagnosis, grade, lymph node status, tumor size, and treatment with surgery remained significant with regard to DSS. Male patients with localized or distant disease had a similar DSS risk during the study period when compared with female patients. Compared with patients with EAC histology, patients with ESCC histology had a worse DSS risk (HR = 1.16; P=0.002) among patients with localized disease and a better DSS risk (HR=0.87; P=0.001) among patients with distant disease. Compared to NHW patients, the black patients had a lower DSS among patients with regional (HR = 1.2; P<0.0001) and distant (HR = 1.2; P<0.0001) disease.

**Table 3:** Multivariable cox proportional hazards analysis of clinicopathologic factors associated with death from esophageal cancer

Factor	HR	P	95% CI	
Sex				
Female	Referent			
Male	1.08	0.002	1.03	1.12
Tumor grade				
I	Referent			
II	1.25	<0.0001	1.14	1.38
III	1.59	<0.0001	1.45	1.74
Undifferentiated	1.42	<0.0001	1.21	1.68
Age at diagnosis, years				
<=65	Referent			
>65	1.12	<0.0001	1.08	1.16
Ethnic group				
NHW	Referent			
Black	1.19	<0.0001	1.12	1.29
HW	1.08	0.03	1.01	1.18
Asian	0.99	0.9	0.92	1.08
Tumor histology				
EAC	Referent			
ESCC	0.94	0.02	0.90	0.99
Lymph node status				
Negative	Referent			
Positive	1.58	<0.0001	1.52	1.68
Cancer-directed surgery				
Not performed	Referent			
Performed	0.45	<0.0001	0.43	0.47
Tumor size (cm)	1.02	<0.0001	1.019	1.03
Tumor location within esophagus				
Upper	Referent			
Middle	1.08	0.02	1.01	1.16
Lower	1.06	0.08	0.99	1.14
Year of diagnosis				
1998-2005	Referent			
2006-2013	0.92	<0.0001	0.89	0.96

HR, hazard ratio; CI, confidence interval; EAC, esophageal adenocarcinoma; ESCC, esophageal squamous cell carcinoma.

**Table 4:** Multivariable cox proportional hazards analyses of clinicopathologic factors associated with death from esophageal cancer, stratified by SEER summary stage

Factor	Localized(n=11,548)				Regional(n=15,431)				Distant(n=17,020)			
	HR	P	95% CI		HR	P	95% CI		HR	P	95% CI	
Sex												
Female	Referent											
Male		NS	0.9	1.2	1.1	0.02	1.02	1.2		NS	1.0	1.2
Tumor grade												
I	Referent											
II	1.3	0.002	1.1	1.6	1.2	0.008	1.1	1.4	1.1	0.5	0.9	1.2
III	1.7	<0.0001	1.4	2.0	1.4	<0.0001	1.3	1.7	1.2	0.01	1.0	1.5
Undifferentiated	1.8	0.001	1.3	2.6	1.3	0.1	1.0	1.6	1.2	0.3	0.9	1.5
Age at diagnosis, years												
<=65	Referent											
>65	1.3	<0.0001	1.2	1.5	1.2	<0.0001	1.1	1.3	1.16	<0.0001	1.1	1.2
Ethnic group												
NHW	Referent											
Black	1.1	0.2	1.0	1.3	1.2	<0.0001	1.1	1.3	1.2	<0.0001	1.1	1.3
HW	1.1	0.3	0.9	1.3	1.0	0.8	0.9	1.1	1.1	0.1	1.0	1.2
Asian	0.8	0.08	0.7	1.0	1.1	0.3	0.9	1.2	1.0	0.5	0.8	1.1
Tumor histology												
EAC	Referent											
ESCC	1.16	0.002	1.1	1.3		NS	0.9	1.0	0.87	0.001	0.8	0.9
Lymph node status												
Negative	Referent											
Positive	1.1	<0.0001	1.1	1.2	1.4	<0.0001	1.2	1.3	1.14	<0.0001	1.1	1.2
Cancer-directed surgery												
Not performed	Referent											
Performed	0.4	<0.0001	0.3	0.5	0.6	<0.0001	0.6	0.7	0.55	<0.0001	0.5	0.6
Tumor size (cm)	1.02	<0.0001	1.02	1.03	1.02	<0.0001	1.02	1.03	1.01	<0.0001	1.004	1.02
Tumor location within esophagus												
Upper	Referent											
Middle		NS	0.9	1.2		NS	0.9	1.1	1.1	0.03	1.02	1.3
Lower		NS	0.8	1.0		NS	0.9	1.1	1.0	0.4	0.9	1.2
Year of diagnosis												
1998-2005	Referent											
2006-2013	0.89	0.02	0.8	0.98	0.88	<0.0001	0.8	0.9	0.9	0.008	0.9	0.98

HR, hazard ratio; CI, confidence interval; NS, not significant; EAC, esophageal adenocarcinoma; ESCC, esophageal squamous cell carcinoma.

Stratification analyses based on histological types were also performed (Table 5). Some correlative factors that influenced the prognosis co-existed in different histology types, such as tumor grade, age, lymph node status, cancer directed surgery, and tumor size. Surgery was a prominent favorable factor to the DSS of the patients, whether in EAC (HR=0.41; P<0.0001) or ESCC cohorts (HR=0.6; P<0.0001). It should be noted that some factors showed differences with the two histology types. In the EAC cohort, the HRs were similar in all sex and ethnicity categories. In contrast, distinctions were apparent in the ESCC cohort; male sex and black patients were unfavorable factors for DSS. However, the tumor location appeared to have no effect on the HRs in the two cohorts.

**Table 5:** Multivariable cox proportional hazards analyses of clinicopathologic factors associated with death by esophageal cancer, stratified by tumor histology

Factor	EAC (n = 30,705)				ESCC(n =19,061)			
	HR	P	95% CI		HR	P	95% CI	
Sex								
Female	Referent							
Male		NS	1.0	1.1	1.13	<0.0001	1.1	1.2
Tumor grade								
I	Referent							
II	1.3	<0.0001	1.1	1.4	1.3	<0.0001	1.1	1.5
III	1.7	<0.0001	1.6	2.0	1.4	<0.0001	1.3	1.6
Undifferentiated	1.6	<0.0001	1.4	2.0	1.2	0.2	0.9	1.6
Age at diagnosis, years								
<=65	Referent							
>65	1.11	<0.0001	1.1	1.2	1.1	<0.0001	1.05	1.2
Ethnic group								
NHW	Referent							
Black		NS	0.9	1.2	1.2	<0.0001	1.1	1.3
HW		NS	1.0	1.2	1.1	0.2	1.0	1.2
Asian		NS	0.8	1.1	1.0	0.9	0.9	1.1
Lymph node status								
Negative	Referent							
Positive	1.38	<0.0001	1.3	1.4	1.19	<0.0001	1.1	1.2
Cancer-directed surgery								
Not performed	Referent							
Performed	0.41	<0.0001	0.4	0.43	0.6	<0.0001	0.55	0.63
Tumor size (cm)	1.02	<0.0001	1.02	1.03	1.02	<0.0001	1.02	1.03
Tumor location within esophagus								
Upper	Referent							
Middle		NS	1.0	1.3		NS	1.0	1.1
Lower		NS	0.9	1.2		NS	1.0	1.2
Year of diagnosis								
1998-2005	Referent							
2006-2013		NS	1.0	1.1		NS	0.9	1.1

HR, hazard ratio; CI, confidence interval; NS, not significant; EAC, esophageal adenocarcinoma; ESCC, esophageal squamous cell carcinoma.

**Figure 4:**
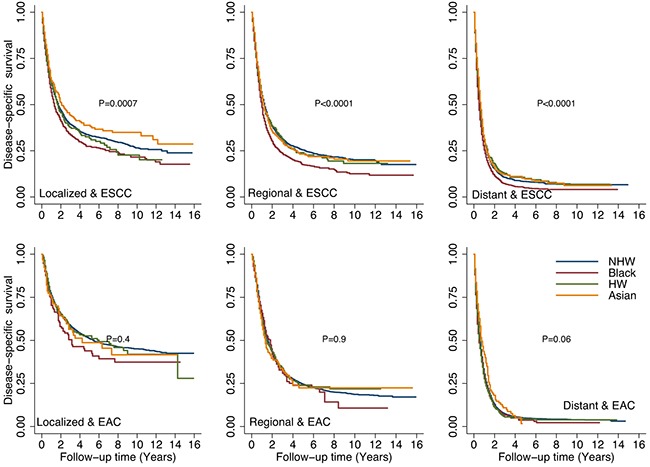
Comparison of disease-specific survival (DSS) rates by different racial/ethnic groups adjusted by stage and tumor histology

When adjusted for stage and tumor histology (Figure 4), the DSS rates were significantly lower for black patients than for other racial/ethnic groups in patients with ESCC (localized, regional and distant disease). There were no significant differences among racial/ethnic groups in patients with EAC (any stage). When we looked at the overall survival, we found the similar trends about racial/ethnic groups impact as in DSS (data not shown).

## DISCUSSION

Our study represented a relatively comprehensive population-based analysis of esophageal cancer that included 49,766 patients. Different from previous SEER data analyses [13–15], we added data in recent years and performed in-depth analysis about the relationship between histology and race/ ethnicity. More interactions between prognostic factors were analyzed here. We found that racial differences existed in incidence rates, prognosis, and risk factors. Consistent with a prior study [1], the study showed significant differences in tumor characteristics and incidence on the basis of ethnicity.

The different trends in the ethnic/racial groups suggested that racial difference existed in the epidemiology and the etiology of ESCC and EAC. The trends in esophageal cancer incidence by race/ethnicity during the period of 1973 to 1998 [16] and the epidemiology in different racial groups from 1991 to 2000 [17] based on SEER data were reported previously. Updated data were given in our results from 1973–2013 and different trends of incidence in males and females among ethnic groups were observed. The incidence trend of ESCC and EAC had geographical variation. The USA and a few European countries had the decreased ESCC incidence, accompanied by the increase of EAC [18]. We performed the further in-depth analysis about the racial differences in incidence trends. The incidence of esophageal cancer decreased in all subgroups and most dramatically in black males group. The curves of incidence in ESCC and EAC in different racial groups were not consistent. We found that black males groups had the significant decreased incidences in ESCC and the slightly increased incidences in EAC. By contrast, White males had increased incidences of EAC and decreased incidences of ESCC since 1973. These differences suggested the incidence rate trends of the ethnic groups depended on the distribution of the histology types. Previous data showed that the overall incidence of ESCC in the United States declined from 3.0 per 100,000 to 2.1 per 100,000 subjects from 1973–2002, and was observed in all race and sex groups. The incidence of EAC increased at the same time, which was most dramatically in Caucasian males [19]. Some studies reported that the incidence of EAC among white males in the United States increased from 0.5–0.9/100,000 in the 1970s, to 3.2–4.0/100,000 in the next two decades [20] and slowed down since 1996 [21], but did not change in the Asian population [8]. The incidence of EAC increased over the same time period for African Americans, while the incidence of ESCC, the predominant histological type for this group, decreased [22]. Our results were similar, but especially emphasized the decline of ESCC in black patients and the increase of EAC in white patients, which were the most dramatic changes among the racial groups. More recent data from 1973–2013 were analyzed here. These analyses indicated the race factor was important in the etiology of different histological types of this cancer.

The phenomenon of differing incidence by histological type byethnicity could be explained by the change in the commonly reported risk factors. Based on previous studies, the rise of obesity [23], the increase in gastroesophageal reflux [24], and the decline of *Helicobacter pylori* infection [25] in NHW may be related to the increased EAC incidence. The proportions of Barrett esophagus [26] and erosive reflux disease [27] are higher among the NHW population, and have been increasing, and are major known risk factors risk of EAC. As for ESCC, the lower level of smoking and alcohol consumption in blacks might have been related to the decreased incidence rates [28]. Apart from these reported factors, the disease characteristics of ESCC and EAC also differed a lot. In our work, distribution of ESCC and EAC was different both in terms of tumor location and year of diagnosis. Significant relationship between histology and tumor location were found in previous study [29]. EAC always occurred in the lower third of the esophagus and originated predominantly from the Barrett esophagus [4], while ESCC occurred mostly in the upper two-thirds of the esophagus [30]. The previous reports and our analyses together, indicated that different histological types had their specific disease characteristics and risk factors. Living habits and environments varied among races/ethnicities and these could explain the changes of incidence.

The ethnic differences observed in our study may have contributions to the survival differences. Conditional survival for esophageal cancer reported previously showed improved prognosis from 1988 to 2011 especially in patients with advanced disease [31]. Although the improving prognosis was encouraging to patients and clinicians, different survival existed in racial groups and the reasons for these should be considered. In the total cohort, black patients showed the lowest DSS among the four ethnic/racial groups, while NHW patients had the best survival. ESCC was determined to be a favorable factor for DSS. Although most individuals in the black and Asians groups were diagnosed with ESCC, as described above, black patients still had a lower DSS, indicating that some other factors should be considered regarding prognoses. The location has been reported to be an unfavorable factor or an unrelated factor for prognosis of the disease [30, 32, 33]. However, our analyses showed that the middle part of the esophagus, but not the lower part, was associated with a poorer DSS (HR=1.08; P=0.02). Regarding the diagnosis year, our results showed that after 2005 was a favorable factor for DSS, especially in the regional disease. Although the SEER data did not reflect the specific therapy of the patients, we speculated that it was related with the effective systemic treatment or clinical therapy in recent years [34, 35]. From another analysis from SEER database, diagnosis at a localized stage increased significantly from 1973 to 2009, as well as the surgical treatment and adjuvant radiation therapy [13]. Black patients, however, were diagnosed mostly as ESCC before 2005 and probably had less opportunity for treatments. This could be a reasonable explanation for the poorer DSS in black patients. Surgery remains the primary curative modality and improvements in surgery methods promoted the survival of the patients [36]. In our data analyses, black patients had less tumor directed surgery than those in the other ethnic groups. In a conclusion, mid-esophagus disease, diagnosed before 2005, and had no directed surgery might explain the worse survival of black patients versus those in the other groups.

The survival of esophageal cancer patients differed when compared within different stage subgroups. From prior evidence, the prognosis of esophageal cancer varied significantly with the stage at diagnosis [37]. Esophageal cancer patients in Germany, with localized cancer stages, had a better prognosis than did those with distant stages (5-year relative survival, 44.4% versus 7.3%, respectively) [38]. A population based analysis showed differences in clinical outcome between different racial groups with locoregional esophageal cancer and the Black groups with locoregional esophageal cancer had poorer survival [39]. Many factors, such as lymph node status, tumor location, and tumor grade, should be taken into consideration when identifying the cancer stage. In our stratified analyses, black patients had a significantly poorer DSS if they had regional or distant stage disease. This data confirmed that the difference among ethnic groups existed, especially in regional and distant stage disease; early stage disease did not show any racial differences. When stratified by stage and histology types, no special factor in EAC group was found, and the survival curves were not different among the ethnic groups. Regarding ESCC, black patients showed unfavorable factors for DSS regardless of the tumor stage. These findings pointed out that ESCC and EAC had different biological behaviors depending on ethnicity. Genetic variations between racial groups should be considered. Some genes like GSTP1 was related with ESCC in the South African population [40]; high CYP3A5 activity could increase esophageal cancer risk in Black Africans [41]. Genetic variants associated with ESCC in Chinese, Indians or other populations were reported [42–44]. Programmed death ligand-1 (PD-L1) was reported as a potential biomarker for the prognosis of ESCC, and the high expression indicated a poor prognosis [45, 46]. But there was not a uniform conclusion about the specific gene biomarkers for different ethnicities in esophageal cancers. The genetic diversity existed among the ethnic groups may have a role in the etiology and survival of ESCC and EAC.

Although esophagectomy is considered the standard treatment for resectable disease, the cure rates are still limited (25%–35%) [47]. We found that cancer-directed surgery rates were generally low. The surgery percentage was lowest, only 14.4%, for black patients. Definitive chemo-radiation is recommended for cervical esophageal cancer (upper location), while esophagectomy or preoperative chemo-radiation is recommended for non-cervical esophageal cancer (middle and lower locations). Based on the results described above, the limitation of the tumor location resulted in the loss of the chance to operate. The patients in the black and Asian groups were characterized with mainly ESCC. The relationship between tumor location and racial group described above could have affected the proportion of each group that underwent surgery.

This study has several limitations. First, selection bias was inevitable because this was a retrospective population-based study. Second, data for some factors, including tumor grade, SEER summary stage, and tumor location, were not known for all patients. This may have influenced the comparison of the subgroups with the total population. Third, informations about chemotherapy were not included. However, our study comprehensively analyzed the racial/ethnic differences in esophageal cancer, the incidence patterns in recent years, and additional related factors. The potential relationships of the prognostic factors, such as ethnicity, histology, and location are complex and could be significant. Our findings may help to understand the tumor biological behavior and could identify esophageal cancer high risk groups in clinical practice.

In conclusion, the clinical characteristics of esophageal cancer have changed in recent years. We found that ethnic and racial groups differed significantly in incidence rates, prognoses, and risk factors. The racial/ethnic differences were reflected in many factors such as histological type, tumor location, and cancer stage. Racial/ethnic differences may be the result of genetic diversity and differences in lifestyle and risk factors for the disease. We determined that the racial/ethnic differences were correlated with factors such as histological type, tumor location, and cancer stage, resulting in the different trends for survival. The histological type distribution between racial/ethnic groups could be an important consideration concerning the incidence and the survival trend. Clinicians should consider esophageal cancer as a heterogeneous disease. Prognostic factors such as tumor location, sex, and histology could affect the disease to varying degrees in specific patient groups. When making clinical decisions for a patient, all of these aspects should be considered.

## MATERIALS AND METHODS

### Patient selection and data collection

The methods for this study overlap those used in our previous analysis of ethnic differences in lung and bronchial cancer [48]. The data were obtained from all 18 U.S. cancer registries in the Surveillance, Epidemiology, and End Results (SEER) Program database (National Cancer Institute) by using the SEER*Stat software program (version 8.3.2; http://seer.cancer.gov/seerstat [accessed June 28, 2016]) under a data user agreement. Patient records were anonymized and de-identified prior to analyses. Because the data were de-identified and from a third party, no ethics committee review approval was needed. The SEER database was searched to identify patients whose primary tumor sites were coded as C15.0–C15.9 (esophagus) and whose cancers were diagnosed from 1998 to 2013. We included only histological codes for adenocarcinomas (8140–8573) and squamous cell cancers (8050–8082). We excluded 266 patients with race coded as American Indian/Alaska Native because of the small numbers; 156 patients with race coded as unknown were also excluded. From the SEER database, we extracted data on patient demographics, primary tumor site, tumor morphology, cancer stage at diagnosis, first course of treatment, and follow-up vital status. The geographical scope of the SEER database was described previously [49–51]. The SEER*Stat program was used to calculate incidence rates and trends over time. Cancer incidence rates for esophageal cancer were presented for each subtype (EAC&ESCC) by race and sex. Race groups included white, black, others (from 1973 to 2013) and white, black and Asian and Pacific Islander (from 2000-2013). All incidence rates were calculated per 100,000 person-years and were age-adjusted to the 2000 US standard population.

### Statistical analysis

Our primary interest was esophageal cancer patient ethnicity. The patients were categorized into four broad groups: NHW, black, HW, and Asian/Pacific Islander. The chi-squared test and Kruskal-Wallis test were used to assess differences in patient characteristics, management, and outcomes among the four groups.

The primary endpoint of this study was disease-specific survival (DSS), which was defined as the number of years from the date of esophageal cancer diagnosis to the date of cancer-related death, date last known to be alive, or November 30, 2013, whichever came first. DSS curves for the study patients were calculated using the Kaplan-Meier method. Patients who died during follow-up or survived beyond November 30, 2013, were censored. Multivariable Cox proportional hazards models were used to determine the influence of patient, tumor, and treatment factors of known or potential prognostic value (age at diagnosis, sex, year of diagnosis, ethnicity, cancer stage, tumor grade, primary tumor site, and primary surgery) on DSS. The Stata/SE software program (version 12; StataCorp, College Station, TX, USA) was used for statistical analyses. All tests were two-tailed, and statistical significance was set at P<0.05.

## Abbreviations

EAC: esophageal adenocarcinoma
ESCC: squamous cell carcinoma
NHW: non-Hispanic white
HW: Hispanic white
SEER: Surveillance, Epidemiology, and End Results
NAACCR: North American Association of Central Cancer Registries
DSS: disease-specific survival
OS: overall survival
HR: hazard ratio
CI: confidence interval
NS: not significant
